# Beneficial Relationships Between Endophytic Bacteria and Medicinal Plants

**DOI:** 10.3389/fpls.2021.646146

**Published:** 2021-04-22

**Authors:** Wei Wu, Wenhua Chen, Shiyu Liu, Jianjun Wu, Yeting Zhu, Luping Qin, Bo Zhu

**Affiliations:** School of Pharmacy, Zhejiang Chinese Medical University, Hangzhou, China

**Keywords:** bacterial community, endophytic bacteria, medicinal plants, plant growth-promoting bacteria, plant-microbe relationships, secondary metabolites

## Abstract

Plants benefit extensively from endophytic bacteria, which live in host plant tissues exerting no harmful effects. Bacterial endophytes promote the growth of host plants and enhance their resistance toward various pathogens and environmental stresses. They can also regulate the synthesis of secondary metabolites with significant medicinal properties and produce various biological effects. This review summarizes recent studies on the relationships between bacterial endophytes and medicinal plants. Endophytic bacteria have numerous applications in agriculture, medicine, and other industries: improving plant growth, promoting resistance toward both biotic and abiotic stresses, and producing metabolites with medicinal potential. Their distribution and population structure are affected by their host plant’s genetic characteristics and health and by the ecology of the surrounding environment. Understanding bacterial endophytes can help us use them more effectively and apply them to medicinal plants to improve yield and quality.

## Introduction

The quality and yield of medicinal plants are significantly influenced by environmental factors, such as temperature, illumination, moisture, soil conditions, and the presence and identity of soil fauna (Namdar et al., 2019). It is increasingly recognized that medicinal plants can also be strongly influenced by their relationships with specific bacterial endophytes (Ek-Ramos et al., 2019). Long-term, symbiotic relationships between host plants and endophytes can promote the growth of plants and be especially useful in agricultural practice (Compant et al., 2010).

Bacterial endophytes are present in the flowers, leaves, roots, seeds, and stems of plants (Qin et al., 2009; Compant et al., 2011; Elmagzob et al., 2019). Bacterial colonization occurs at particular stages of plant development, and even at the seed stage, there may already be a stable endophytic bacterial community (Mocali et al., 2003). These endophytes use the plant’s internal environment (i.e., the endosphere) as a unique niche to protect themselves from drastic altered external environments (Senthilkumar et al., 2011). The evolution of these highly specialized symbioses requires tight coordination of physiology, structure, and life cycles between the partner organisms (Saikkonen et al., 2004; Reinhold-Hurek and Hurek, 2011), and the resulting partnership benefits both species (Aravind et al., 2010). Such mutualisms serve essential functions in terrestrial ecosystems: host plants house and protect the endophytes, which in turn promote the growth of plants via nitrogen fixation, phosphorus enrichment, and the synthesis of phytohormones (Qin et al., 2015; Borah et al., 2019). Moreover, pathogenic microorganisms and endophytic bacteria occupy the same niche within plants, and inoculation with endophytic bacteria is a suitable method to biologically control pathogens (Senthilkumar et al., 2011). Medicinal plants-isolated bacterial endophytes can produce bioactive metabolites and to significantly induce the secondary metabolite production by their host plants. The host-endophyte relationship can be regarded as a flexible, dynamic interaction, in which endophytic bacteria alter their gene expression or produce different metabolites based on small changes in host plant growth and vice versa (Ek-Ramos et al., 2019). Despite their importance to the plant micro-ecosystem, bacterial endophytes’ relationship to their host plants remains poorly understood (Tiwari et al., 2013).

The community of bacterial endophytes is influenced by both biotic and abiotic factors, which shape their species composition, community structure, diversity, and functions (Walitang et al., 2018). Environmental factors not only affect the distribution of a medicinal plant, but also determine the species of bacterial endophyte which can colonize host plant throughout its life cycle (Deng et al., 2011). Endophyte diversity is also influenced by host plant characteristics, including genotype (Walitang et al., 2018), tissue (Dai et al., 2014), growth stage (age) (Vendan et al., 2010), and health status (Bogas et al., 2015). The non-random distribution of endophytic bacterial species thus provides clues as to their biology and ecology. However, there is limited information on the plant and environmental factors that shape endophytic bacterial community structure and how endophytes regulate their hosts by synthesizing primary and secondary metabolites.

Here, we summarize the external factors that affect endophytic bacteria, and we survey the potential uses of them in growth promotion, pathogen resistance, and secondary metabolism of medicinal plants. Understanding and using these symbiotic relationships can enable us to more effectively cultivate valuable plants for human use and improve the quality and yield of medicinal materials.

## Factors That Affect Endophytic Bacteria: Environment and Host Plant

### Environmental Effects on Community Structure of Endophytic Bacteria

The community structure of endophytic bacteria is affected by multiple external factors, including season, altitude, latitude, longitude, and soil conditions (Chiellini et al., 2014; Yang et al., 2017; Table 1). For example, Actinobacteria and Proteobacteria were the most abundant endophytic bacteria of mulberry (*Morus* L.) in the spring, whereas only Proteobacteria were found in the fall. The spring endophytic bacteria were also characterized by greater diversity and a larger number of species (Shannon, 14.00; Simpson, 0.30; Chao, 1018) compared with autumn endophytes (Shannon, 6.62; Simpson, 1.26; Chao, 654) (Ou et al., 2019). Another study revealed that the *Pyrus ussuriensi* community structure substantially correlated to the carbon, nitrogen, pH, and temperature of soil. In particular, the richness and diversity of root endophytic bacteria increased with increased nitrogen content (Yarte et al., 2020). Xu et al. (2014) reported that soil water content and annual precipitation were strongly correlated with the endophytic bacterial RFLP (restriction fragment length polymorphism) type variation in *Caragana jubata* and *Oxytropis ochrocephala*, followed by latitude, longitude, soil nitrogen content, and soil potassium content (Xu et al., 2014). Bacterial endophyte community structure changes with changing environmental conditions and the maintenance of a shifting and diverse endophytic community may form part of the physiological strategy by which plants adapt to their environment.

**TABLE 1:** Factors affecting the community structure of endophytic bacteria in medicinal plants.

Habitat	Representative host plant	Isolated part(s)	Factor(s)	Factor(s) explanatory comments	References
Mountains in subtropics	*Caragana jubata*	Root	Environment: altitude	Different dominant endophytic bacteria	Xu et al., 2014
Mountains in subtropics	*Stellera chamaejasme*	Leaf, stem, and root	Tissue	The OTUs number of endophytic bacteria from high to low in different tissues were leaf > stem > root	Jin et al., 2014
Karst landform	*Cyrtomium fortunei*	Root	Environment: soil type	The highest endophyte numbers were observed in low calcium soil	Li F. et al., 2019
Grassland habitat in savanna	*Baccharis dracunculifolia*	Root and leaf	Tissue	The OTUs number of endophytic bacteria from high to low in different tissues were root > leaf	Santana et al., 2016
Plantation	*Paullinia cupana*	Leaf	Health status of plants	Lower relative abundance in healthy plants than in susceptible plants	Bogas et al., 2015
Temperate maritime climate islands	*Pseudowintera colorata*	Leaf, stem and root	Tissue age	The species richness of endophytic bacteria increased with tissue age	Purushotham et al., 2020
Temperate forest	*Cinnamomum camphora*	Leaf	Season	The order of the endophytes richness in the samples was spring > summer > early winter	Elmagzob et al., 2019
Subtropical region	*Morus* sp.	Branch	Season	Spring samples harbor higher bacterial OTUs, α-diversity, and bacterial community complexity than autumn samples	Ou et al., 2019
Mediterranean region	*Helianthus annuus*	Root	Environment: moisture	Endophyte colonization was positively correlated with humidity	Santos et al., 2014
Subtropical botanical gardens	*Sarracenia* spp.	Rhizome	Taxonomy of plants	Different dominant endophytic bacteria	Sexton et al., 2019

### Host Plant Effects on Endophytic Bacteria

The relationship between host plants and specific endophytic bacterial isolates may exhibit characteristics of either parasitism or mutualism, depending on host genotype, tissue, and health status (Cope-Selby et al., 2017), and multiple studies showed that endophytic bacterial communities are considerably affected by their host plants. A study in the Patagonian ecosystem showed that bacterial abundance and diversity were higher on *Hieracium pilosella* than on *Gaultheria mucronata* (Zhang et al., 2019), a result consistent with the assumption that the diverse physiological structures, metabolites, and growth habits of different plants affect their ability to recruit various endophytic bacteria (Kawaguchi and Minamisawa, 2010; Campisano et al., 2014). Host plant health status also influences endophyte colonization: the bacterial endophytic community of *Paullinia cupana* with asymptomatic anthracnose comprised mainly Firmicutes, whereas that of plants with symptomatic anthracnose comprised mainly Acidobacteria (Bogas et al., 2015). Such results may reflect pathogen-mediated surface damage to the host, promoting the establishment of some endophytes and disrupting the original stable microbial ecological environment (Hallmann et al., 1998). In one study, PCR-based molecular techniques were employed to investigate the cultivable bacteria isolated from the leaf, root, and stem compartments of *Echinacea angustifolia* (DC.) Hell and *Echinacea purpurea* (L.) Moench, which demonstrated that these two medicinal plants and the respective compartments possessed different types of bacterial communities, suggesting the strong selective pressure and the low-degree strain sharing in the plant tissues (Chiellini et al., 2014). These differences might be attributed to differential nutritional and environmental conditions to which the aerial parts and the roots of the plants were exposed, or due to the phytochemical and anatomical features, which in turn established particular ecological niches for the endophytes. Endophytic bacteria are possibly selected based on their strategies for adaptation and the tolerance under differential conditions in a variety of plant compartments. The differences in the medicinal properties can be explained by the presence of distinct bacterial communities in differential plant species and among the compartments of a single plant species. In general, endophytic bacteria can adjust their structure and diversity in response to different plant genotypes, organs, health statuses, and growth stages in order to obtain a consistent supply of nutrients. Moreover, in an olive system, it was found that belowground communities of endophytic bacteria were mainly determined by the cultivar genotype grown under same agronomic, environmental, and pedological conditions, indicating that the plant genotype serves as a main factor to shape the belowground bacterial communities of olive (Fernandez-Gonzalez et al., 2019).

## Beneficial Effects of Endophytic Bacteria on Host Plants

### Collected Medicinal Plants and the Associated Endophytic Bacteria

**FIGURE 1:**
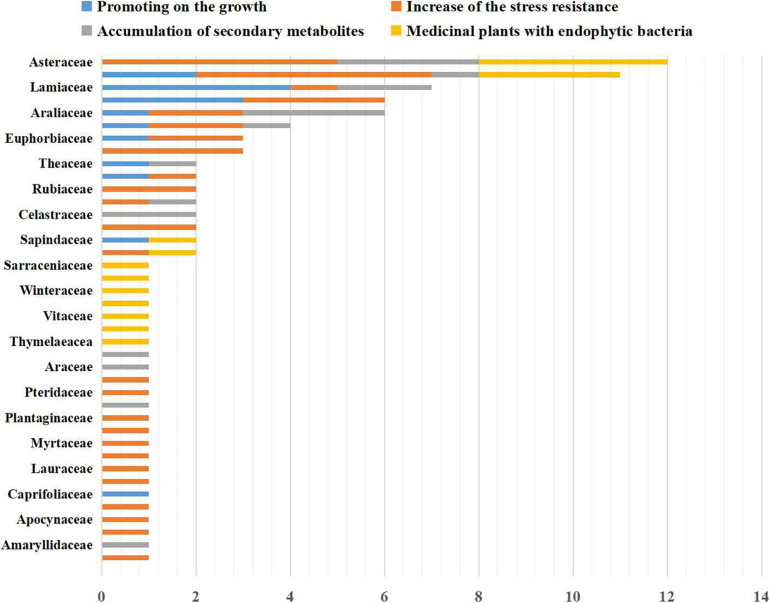
Taxonomy of the 86 species of medicinal plants included in the survey and reference analysis (*x*-axis: number of species in the family; *y*-axis: type of family).

**FIGURE 2:**
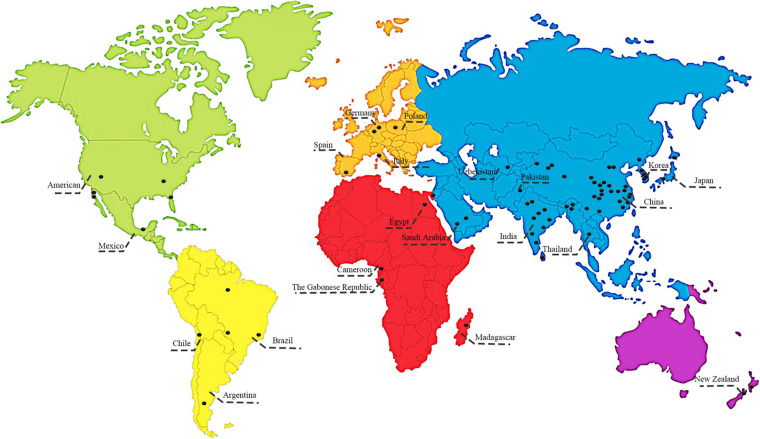
Geographical distribution of medicinal plants and related endophytic bacteria, considered in the survey.

Our survey and analysis have documented the presence of mutually beneficial relationships between bacterial endophytes and 86 medicinal plants from 40 families (Figure 1). Surveyed plants included Amaranthaceae (2 taxa), Amaryllidaceae (1 taxon), Anacardiaceae (1 taxon), Apiaceae (4 taxa), Apocynaceae (1 taxon), Araceae (1 taxon), Araliaceae (2 taxa), Asteraceae (11 taxa), Berberidaceae (1 taxon), Brassicaceae (2 taxa), Cactaceae (1 taxon), Caprifoliaceae (1 taxon), Celastraceae (2 taxa), Chenopodiaceae (3 taxa), Dryopteridaceae (1 taxon), Euphorbiaceae (4 taxa), Fabaceae (11 taxa), Ginkgoaceae (1 taxon), Lamiaceae (7 taxa), Lauraceae (2 taxa), Liliaceae (2 taxa), Meliaceae (1 taxon), Moraceae (2 taxa), Myrtaceae (1 taxon), Orchidaceae (1 taxon), Plantaginaceae (1 taxon), Poaceae (4 taxa), Polygonaceae (1 taxon), Pteridaceae (1 taxon), Rosaceae (1 taxon), Rubiaceae (2 taxa), Sapindaceae (2 taxa), Sarraceniaceae (1 taxon), Saururaceae (1 taxon), Theaceae (2 taxa), Thymelaeaceae (1 taxon), Ulmaceae (1 taxon), Vitaceae (1 taxon), Winteraceae (1 taxon), and Zingiberaceae (2 taxa). As shown in Figure 2, the medicinal plants we surveyed were mainly distributed in Asia and Europe; there were a few studies on medicinal plant endophytes from North America and Australia, but none from extreme regions. Future research should focus on endophytic bacteria and medicinal plants in areas where there has been relatively less research. A total of 11 orders and 88 genera of endophytic bacteria had documented associations with medicinal plants in the literature. The most common orders were Bacillales, Enterobacterales, and Pseudomonadales, which accounted for 72.62%. The most common genera were *Bacillus*, *Pantoea* and *Pseudomonas*, which accounted for 58.92%. *Streptomyces* are widely reported as promoting the growth and development of plants, and *Bacillus*, *Pseudomonas* and *Paenibacillus* can influence the growth, stress resistance and metabolism of medicinal plants (Gao et al., 2015; Zhao et al., 2015; Qi et al., 2021). Based on the information collected above, we suggest that future studies prioritize exploring these beneficial endophytic bacteria. However, such endophytic bacteria mentioned in the literature have been isolated by culture techniques. Our present understanding of medicinal plant endophytes derives almost entirely from culture-based diversity analyses (Liu et al., 2017), though most of the environmental bacteria are unculturable. This constraint limits our understanding of medicinal plant endophytic bacteria and the influence of plant hosts on the structure of bacterial community: the impact of unculturable bacteria on host plant responses should focus on future research.

### Promotion of Host Plant Growth

Endophytic bacteria are potential candidates to promote the growth of medicinal plants, such as to enhance root and shoot biomass and stimulate seed germination (Vendan et al., 2010). Previously, they were reported to increase the growth of plants through the synthesis of indole acetic acid (IAA) (Table 2; Fouda et al., 2021). In particular, microbial synthesis of IAA by the tryptophan-dependent pathway can affect the growth of plants (Gravel et al., 2007). However, the underlying mechanisms have primarily been studied *in vitro*, and future studies need to investigate specific metabolites and mechanisms of growth promotion during the actual interaction between the endophytic bacterium and its host. One bacterial endophyte, *Serratia marcescens* AL2-16, can fix nitrogen in *Achyranthes aspera* by capturing atmospheric N and converting it into an available nitrogen form through enzymatic reduction (Devi et al., 2016). Wheat seedlings inoculated with a *Paenibacillus ehimensis* strain from *Lonicera japonica* showed marked increases in growth associated with greater photosynthetic carbon uptake and light use efficiency (Zhao et al., 2015). Furthermore, multiple other benefits on the growth of plants that are attributed to endophytes include modification of root morphology, osmotic adjustment, phosphate siderophore production improvement, solubilization activity enhancement, and stomatal regulation (Compant et al., 2005; Chen et al., 2016; Zhu, 2019). These plant growth-promotive bacterial endophytes are presently used for forest regeneration and contaminated soil phytoremediation (Ryan et al., 2008; Huang et al., 2020). However, these studies only applied single bacterial strains. The combined effects of endophytic bacterial population should be further studied.

**TABLE 2:** Beneficial relationships between endophytic bacteria and host plants.

Role of beneficial bacteria	Host plant	Endophytic bacteria	Effect(s)	References
Plant growth promotion	*Panax ginseng*	*Micrococcus luteus* and *Lysinibacillus fusiformis*	Enhanced seedling biomass	Vendan et al., 2010
	*Lavandula dentata*	*Variovorax* sp.	Increased adventitious root formation and the rooting capacity of cuttings	Pereira et al., 2016
	*Teucrium polium*	*Bacillus cereus* and *Bacillus subtilis*	Increased root length, weights, and root area	Hassan, 2017
	*Coriandrum sativum*	*Bacillus siamensis*	Increased root length, shoot length, and dry weight	Ibrahim et al., 2019
	*Curcuma longa*	*Bacillus* and *Paenibacillus* spp.	Increased root length, shoot length, and root number	Aswathy et al., 2013
	*Lonicera japonica*	*Paenibacillus* and *Bacillus* spp.	Increased shoot and root length and fresh and dry weight	Zhao et al., 2015
	*Achyranthes aspera*	*Serratia marcescens*	Increased shoot length, fresh shoot and root weight, and leaf area	Devi et al., 2016
Enhanced plant resistance to phytopathogens	*Panax notoginseng*	*Bacillus amyloliquefaciens*	Protection of host plants from phytopathogen infection	Ma et al., 2013
	*Curcuma longa*	*Bacillus* sp.	Induced host disease resistance	Jayakumar et al., 2019
	*Panax ginseng*	*Stenotrophomonas maltophilia* and *Bacillus* sp.	Suppressed pathogen mycelial growth	Hong et al., 2018
	*Centella asiatica*	*Cohnella* sp., *Paenibacillus* sp. and *Pantoea* sp.	Induction of plant defense mechanisms	Rakotoniriana et al., 2013
	*Ginkgo biloba*	*Bacillus amyloliquefaciens*	Produced antibiotics and induced systemic resistance	Yang et al., 2014
	*Epimedium brevicornu*	*Phyllobacterium myrsinacearum*	Depressed the growth of the pathogens	He et al., 2009
Improved plant abiotic stress tolerance	*Limonium sinense*	*Glutamicibacter halophytocola*	Improved tolerance to high NaCl concentration	Qin et al., 2018
	*Catharanthus roseus*	*Achromobacter xylosoxidans*	Increased germination percentage and root weight under saline conditions	Karthikeyan et al., 2012
	*Plantago asiatica*	*Paenibacillus* sp.	Degraded phenanthrene	Zhu et al., 2016
	*Tridax procumbens*	*Paenibacillus* sp.	Relieved plant heavy metal stress	Govarthanan et al., 2016
	*Pteris vittata*	*Agrobacterium* spp. and *Bacillus* spp.	Reduced arsenate to arsenite	Xu et al., 2016
	*Euphorbia milii*	*Citrobacter putida*	Removed airborne benzene	Khaksar et al., 2016
Promotion of plant metabolites accumulation	*Atractylodes macrocephala*	*Pseudomonas fluorescens*	Increased production of sesquiterpenoids	Yang et al., 2019
	*Atractylodes lancea*	*Pseudomonas fluorescens*	Essential oil accumulation	Zhou et al., 2016
	*Panax ginseng*	*Paenibacillus polymyxa*	Induced production of ginsenoside	Gao et al., 2015
	*Ligusticum chuanxiong*	*Bacillus subtilis*	Promoted ligustrazine accumulation	Yin et al., 2019
	*Artemisia annua*	*Pseudonocardia* sp.	Increased artemisinin content	Li et al., 2012
	*Panax ginseng*	*Burkholderia* sp.	Increased ginsenoside Rg3	Fu et al., 2018

### Promotion of Host Plant Abiotic Stress Resistance

Some endophytic bacteria can enhance the resistance of host plants to abiotic stresses, such as heavy metals and salinity (Sheng et al., 2011). Salinity primarily inhibits the growth of plants by lowering soil osmotic potential, forcing the plant to lower its own water potential in an effort to obtain and conserve water. Host plant survival under such conditions may be enhanced by mechanisms such as phytohormone modulation that alleviate osmotic stress impacts (Hasanuzzaman et al., 2014). One experiment demonstrated that the presence of *Achromobacter xylosoxidans* reduced ethylene levels in *Catharanthus roseus* and increased the content of antioxidant enzymes such as ascorbate peroxidase, catalase, and superoxide dismutase under saline conditions (Karthikeyan et al., 2012). Moreover, endophytic bacteria stimulated the growth of plants through increasing the nutrient absorption capacity of rhizosphere and enhancing photosynthesis. In a study of *Cicer arietinum*, inoculation with *Bacillus subtilis* facilitated the synthesis of photosynthetic pigments and enhanced the plants’ contents of calcium, magnesium, nitrogen, and potassium (Abd_Allah et al., 2018). *Silene vulgaris* with *P. helmanticensis* H16, *Proteus vulgaris* H7, or *Pseudomonas* sp. H15 treatment had higher fresh shoot biomass under Cd and Zn stress than controls (Płociniczak et al., 2019). These mechanisms of abiotic stress tolerance are through production of antibiotics, enzymatic and non-enzymatic antioxidants, and phytohormones (Khare et al., 2018). Future studies are needed on the interactions among the environment, host plants, and endophytic bacteria, particularly on mechanisms by which endophytic bacteria help their hosts resist environmental stress.

Plants in xenobiotics-contaminated soil naturally recruit endophytes possessing essential genes to degrade the contaminants (Siciliano et al., 2001). In fact, the genes for degradation of nitro-aromatic compounds were found more prevalent in endophytic strains in the fields with nitro-aromatic contaminations than in rhizospheric or soil microbial communities (Ryan et al., 2008). Barac et al. (2004) described an application of bacterial endophytes possessing substantial biotechnological potentials, who demonstrated that engineered *Burkholderia cepacia* G4 increased plant tolerance toward toluene and decreased the transpiration of toluene to atmosphere (Barac et al., 2004). Newman and Reynolds (2005) summarized the potential advantages of applying endophytic microorganisms for improving xenobiotic remediation, the major of which is the required genetic engineering of a xenobiotic degradation pathway, while gene manipulation is more easily accomplished in bacteria than in plants (Newman and Reynolds, 2005). Moreover, quantitative expression of pollutant catabolic genes in the endophytic populations could serve as a valuable monitoring tool to assess the efficiency of the process of remediation. The unique niche of interior plant environment provides xenobiotic-degradation strains with larger sizes of population attributed to reduced competition. Furthermore, toxic xenobiotics taken up by plants might be degraded in the planta, thereby reducing phytotoxic effects and preventing potential toxicity on herbivorous fauna residing near or on the contaminated sites. Understanding relevant mechanisms that enable the interactions between these endophytic bacteria and the host plants is essential for fully achievement of their biotechnological potentials for various applications. One of the promising areas of future research is to develop endophytic bacteria that enhance the sustainable production of biomass and bioenergy crops as well as soil contaminant phytoremediation.

### Increased Biotic Resistance of Host Plants

Crop pests and diseases are among the most significant causes of economic losses in agriculture, and at least one study has demonstrated a correlation between the bioactive compounds produced by endophytic bacteria and host plant disease tolerance (Bibi et al., 2012). Likewise, certain endophytic bacteria from *Panax ginseng*, termed pathogen antagonists, demonstrate antimicrobial activity against *Botrytis cinerea* and *Cylindrocarpon destructans*, as well as hydrogen cyanide production *in vitro* (Hong et al., 2018). Some *Pseudomonas* spp. can impede the growth of soil pathogens (Paulin et al., 2009). Endophytic bacteria might protect medicinal plants from pathogens through various mechanisms, including displacing them from their ecological niche within plant tissues and producing antibiotics that directly inhibit their growth (Lacava and Azevedo, 2013). In addition, *Bacillus amyloliquefaciens* Fy11 appears to suppress *Phytophthora* blight on pepper indirectly by promoting induced systemic resistance (ISR) (Yang et al., 2014).

There is evidence suggesting that resistant peach cultivars harbor a greater abundance and diversity of bacterial endophytes and more bacterial antagonists of the pathogen *Agrobacterium tumefaciens* bacterial community may constitute an important component of their *A. tumefaciens* resistance (Li Q. et al., 2019). Furthermore, numerous non-medicinal plants take use of endophytic bacterial consortia or the combinations of other microorganisms and/or inhibitors to combat harmful phytopathogens as well as enhance their growth (Nagpal et al., 2020; Sundaramoorthy, 2012). Therefore, further *in vitro* development of endophyte-plant models is essential. As a matter of fact, although several crop microbiota have been investigated in detail for examining their interactions with the respective hosts, medicinal plant model systems are still missing. The complementary information obtained from modern “omics” studies combining with other system biological techniques are crucial for establishing models in predicting and explaining endophyte-mediated processes (Kaul et al., 2016). Additionally, the market-oriented application of biological control agents is essential as well, though a series of issues need repetitive examination, such as the effects of complex external condition alterations, the best application time of endophytes, and the potential pathogenicity of endophytes under condition changes (30). There is a dynamic, intricate intermingling of multiple bacterial species with the host plant, and significant research will be required to fully understand the effects of these complex relationships on host plant disease resistance.

### Increased Bioactive Compound Accumulation in Medicinal Plants

Research on endophytic bacterial contribution to their medicinal host plant metabolism is complicated by the fact that some secondary metabolites may be produced by the combined activity of both the bacteria and the host (Brader et al., 2014). Some endophytic bacteria are known to induce the production of secondary metabolites in medicinal plants (Tiwari et al., 2010). For example, *Bacillus altitudinis* KX230132.1 serves as an effective elicitor that increases ginsenoside concentrations in the valuable medicinal herb, ginseng (Song et al., 2017). Moreover, such elicitors may also participate directly in the biochemical transformation of active ingredients in medicinal plants. Previous work has demonstrated that *Burkholderia* sp. GE 17-7 can convert the major ginsenoside Rb1 into the minor ginsenoside Rg3, which may possess practical importance to develop the antitumor compound ginsenoside Rg3 (Fu et al., 2018). Such biotransformations using endophytic bacteria have significant potential for promoting the accumulation of rare active ingredients in medicinal plants. However, the specific mechanisms by which endophytic bacteria regulate plant physiology and metabolism remain unknown. Likewise, the processes by which they use intermediate compounds of primary and secondary metabolism as nutrients and precursors for producing new compounds or enhancing existing metabolites are poorly understood.

Previous studies have shown that the synthesis of multiple bioactive secondary metabolites, including alkaloids, sesquiterpenes, polyketones, lactones, organic acids, cyclopeptides, flavonoids, and saponins, with novel applications can be accomplished by endophytes present in host plants (Ek-Ramos et al., 2019). Further activity studies revealed that these endophytic bacteria and their host plants share several similar bio-properties such as antimicrobial, anticancer, anti-inflammatory and anti-HIV activities (Table 3; Rustamova et al., 2020). Endophytes are extremely crucial biological resources, the exploration of which in the future can facilitate environmental sustainability, and they can act as unlimited biomolecule sources for various industrial sectors and benefiting human health. Therefore, it is essential to investigate their genomics as well as the integrated metabolism of endophyte-host plant relationship. Furthermore, it is recommended to deduce the biochemical and physiological characteristics of these endophytes at genomic and metabolomic levels, respectively. To date, no database is exclusively available for endophytes and their metabolites, and the building of which is of great importance in providing solutions for all the aforementioned issues.

**TABLE 3:** Secondary metabolites originated from endophytic bacteria in medicinal plants and their bio-properties.

Class	Compounds	Endophytic bacteria	Host plant	Bio-properties	References
Alkaloids	6-Prenylindole	*Streptomyces* sp.	*Allium tuberosum*	Antifungal activity	Singh and Dubey, 2018
	1-Acetyl-β-carboline	*Aeromicrobium ponti*	*Vochysia divergens*	Antibacterial activity	Gos et al., 2017
	Indole-3-carbaldehyde				
	3-(Hydroxyacetyl)-Indole				
	Brevianamide F				
	3-Acetonylidene-7-Prenylindolin-2-one	*Streptomyces* sp.	*Glycine max*	Antifungal activity	Yan et al., 2014
	7-Isoprenylindole-3-carboxylic acid				
	Vindoline	*Microbacterium* sp.	*Catharanthus roseus*	Treating Hodgkin’s disease and acute leukemia	Anjum and Chandra, 2019
	Camptothecin	*Kytococcus schroeteri*	*Ephedra foliata*	Anticancer activity	Ghiasvand et al., 2019
	2,3-dihydro-2,2-dimethyl-4(1H)-quinazolinone	*Streptomyces* sp.	*Lychnophora ericoides*	Anticancer activity	Conti et al., 2016
	Indole-3-acetic acid	*Pseudomonas fluorescens*	*Atractylodes lancea*	Promoting plant root development and carbohydrates provide	Zhou et al., 2018
	Berberine	*Microbacterium* and *Burkholderia*	*Coptis teeta*	Anti-inflammatory, anti-tumor, and lowering blood sugar activities	Liu et al., 2020
Sesquiterpenes	Xiamycin	*Streptomyces* sp.	*Bruguiera gymnorrhiza*	Anti-HIV activity	Ding et al., 2010
	Trichodones A-C				
	Guignarderemophilanes A-E	*Guignardia mangiferae*	*Gelsemium elegans*	Anti-inflammatory activity	Liu et al., 2015
Polyketones	Grignard dene A	*Guignardia mangiferae*	*Gelsemium elegans*	Anti-inflammatory activity	Liu et al., 2015
	Grignard lactone A				
	Naphthomycins A, D, E, L, K, O-Q	*Streptomyces* sp.	*Maytenus hookeri*	Antimicrobial activity	Yang et al., 2018
Lactones	Cedarmycin A	*Streptomyces* sp.	*Aucuba japonica*	Antifungal and antibacterial activities	Sasaki et al., 2001
	Cedarmycin B				
	Daunorubicin	*Paenibacillus polymyxa*	*Ephedra foliata*	Anticancer activity	Ghiasvand et al., 2019
	Hookerolide	*Streptomyces* sp.	*Maytenus hookeri*	Antimicrobial activities	Yang et al., 2018
	24-demethyl-bafifilomycin A_2_, Z				
Organic acids	Trans cinnamic acid	*Nocardiopsis* sp.	*Zingiber officinale*	Antimicrobial activity	Sabu et al., 2017
	Benzoic acid				
	phthalic acid	*Bacillus atrophaeus* and *Bacillus mojavensis*	*Glycyrrhiza uralensis*	Antifungal and antibacterial activities	Mohamad et al., 2018
Cyclopeptides	cyclo(L-Tyr-L Pro-L-Phe-trans-4-hydroxy-L-Pro)	*Streptomyces* sp.	*Inula cappa*	Antimicrobial activity	Zhou et al., 2014
	cyclo(L-Phe-trans-4-hydroxy-L-Pro)				
	cyclo(L-Val-L-Tyr)				
	Halobacillin	*Streptomyces* sp.	*Bruguiera gymnorrhiza*	Anti-HIV activity	Ding et al., 2010
Flavonoids	7-Methoxy-3,3′,4′,6-tetrahydroxyflavone	*Streptomyces sp.*	*Boesenbergia rotunda*	Anticancer activity	Taechowisan et al., 2014
	2′,7-Dihydroxy-4′,5′-Dimethoxyisoflavone				
	Fisetin				
Saponins	Ginsenoside Rg3	*Burkholderia* sp.	*Panax ginseng*	Anticancer activity	Fu et al., 2017
	Ginsenoside Rh2	*Agrobacterium* sp.	*Panax ginseng*	Anticancer activity	Yan et al., 2019
Others	Ligustrazine	*Bacillus subtilis*	*Ligusticum chuanxiong*	Treating ischemic vascular related diseases	Yin et al., 2019
	Linfuranone A	*Microbispora* sp.	*Clinacanthus siamensis Bremek.*	-	Indananda et al., 2013
	5,7-Dimethoxy-4-phenylcoumarin	*Streptomyces aureofaciens*	*Zingiber officinale*	-	Taechowisan et al., 2007
	bis (2-ethylhexyl) phthalate	*Bacillus subtilis*	*Thymus vulgaris*	Antimicrobial activity	Abdelshafy Mohamad et al., 2020
	1,3- dimethyl-, p-xylene				
	dibutyl phthalate				
	Tetracosane				
	1- –Heptacosanol	*Nocardiopsis* sp.	*Zingiber officinale*	Antimicrobial activity	Sabu et al., 2017

*‘−’ denotes no useful information found in the study.*

## Conclusion and Perspectives

The genotype, morphology, life history, and health status of medicinal plants can affect the composition, distribution, and structure of their endophytic bacterial community through various physiological mechanisms. Therefore, individual plants have unique endophytic bacteria or bacterial communities, which may inhabit specific plant tissues depending on their roles or preferred niches. On the other hand, the distribution and community structure of bacterial endophytes are also strongly influenced by the external environment (Mitter et al., 2013): temperature, humidity, illumination, and geographic location all determine the distribution of medicinal plants and in turn influence their associated endophytic bacterial species. A better understanding on the influences of the external environment on specific endophytic bacteria will allow us to maximize plant benefits by appropriately modifying external conditions after inoculation.

Bacterial endophytes can promote the growth of plants and protect them from environmental stresses and harmful microorganisms. In return, endophytic bacteria obtain greater access to nutrients and improve their growth (Shi et al., 2010). Endophytic bacteria can stimulate medicinal plant growth through improving seed germination, and indeed this mutualistic association may be necessary for successful germination in some species (Verma et al., 2019). Bacteria applied in biological fertilizer can improve plant nutrition and provide an environmentally sustainable means of improving the growth and yield of plants (Vaishnav et al., 2018). Furthermore, the ability of endophytic bacteria to stimulate both production and accumulation of secondary metabolites lends itself to valuable practical applications (Liu et al., 2015). Inoculation with one or several endophytic bacteria has enormous potentials for enhancing bio-active compound production by medicinal plants (Zhou et al., 2015). Moreover, it may be beneficial to explore the fluctuations in medicinal plant yield and quality caused by environmental factors in order to understand the reasons why certain areas produce Dao-di medicinal plants, which are very high-quality medicinal herbs produced from specific regions and have a long tradition of use and excellent medicinal properties. Given the importance of the endophytic bacteria-medicinal plants interactions, studies of such bacteria may enable the successful development of new areas of natural medicine. Under natural environments, microbial communities with mixed species can exhibit competitive advantages in metabolic complexity, productivity, resistance to invasion, and resource efficiency over monocultures (Karkaria et al., 2021). Being capable of reproducibly and predictably constructing microbial communities for biotechnological or synthetic biological applications would guarantee the application of such advantages. Furthermore, the endophytic associations were studied only in approximately 1–2% of the known plant species (Khare et al., 2018), most of which were the land plants, leaving aquatic plants in lakes, ocean, etc., completely untouched. *In situ similis* culturing and isolation strategy in different plant niches can be used for find more endophytic bacteria (Castronovo et al., 2021).

Research on beneficial bacterial strains has been primarily limited to laboratory studies, and future research should therefore focus more on field experiments and practical applications to obtain higher quality medicinal plants. Moreover, we know little about the mechanisms of endophytic bacteria-medicinal plants interactions. In the coming decades, we recommend several priority topics for additional research: (1) the development of innovative approaches for the separation and cultivation of endophytic bacteria in order to build a functional library of endophytic bacteria and investigate the effects of unculturable endophytes on medicinal plants; (2) studies on the effects of endophytic bacterial communities on medicinal plants; (3) artificial transformation of functional bacteria to give them additional beneficial functions; (4) strategies to establish symbiotic endophyte-host plant associations, and simulation of the symbiotic vs. parasitic relationships between endophytic bacteria and medicinal plants; (5) transmission mode (endophytes are transmitted vertically as well from plant reproductive tissues to the next generation), and (6) explorations of the mechanisms by which Dao-di medicinal materials are formed, with an emphasis on the role of endophytic bacterial community structure.

Plants have evolved through continuous interaction with microbes, and it is evident that endophytic bacteria play significant roles in improving plant survival and adaptation (Singh et al., 2017). This review outlines the biotic and abiotic factors that influence community structure and endophytic bacterial distribution and summarizes the beneficial effects of endophytes on their host plants. Such information provides a foundation for further studies and can be applied to obtain better bioactive materials from medicinal plants.

## Author Contributions

LQ and BZ reviewed and finalized manuscript. WW and WC completed the article writing. JW, SL, and YZ integrated information of tables, analyzed, data, and made pictures. All authors reviewed and approved the manuscript.

## Conflict of Interest

The authors declare that the research was conducted in the absence of any commercial or financial relationships that could be construed as a potential conflict of interest.
